# RBOHC‐Generated ROS Tune MIZ2/GNOM‐Dependent Root Halotropism in *Arabidopsis*

**DOI:** 10.1111/pce.70681

**Published:** 2026-06-19

**Authors:** Amir Cohen, Matan Franko, Yvonne Kiere, Yonatan Wexler, Doron Shkolnik

**Affiliations:** ^1^ Robert H. Smith Institute of Plant Sciences and Genetics in Agriculture, Faculty of Agriculture, Food and Environment Hebrew University of Jerusalem Rehovot Israel

## Abstract

*Arabidopsis thaliana* roots adjust their growth away from saline environments through a tightly localised reactive oxygen signal in the root elongation zone. Disruption of this spatial control leads to exaggerated or misdirected bending, revealing how roots fine‐tune their orientation under salt stress.

Soil salinization, driven by unsustainable irrigation, natural processes, and climate change, creates heterogeneous salt distributions that threaten crop productivity and global food security (FAO 2024). Salinity creates adverse conditions for plant growth, affecting root function and overall plant performance. To cope, roots deploy adaptive strategies, including altered growth rates, lateral root patterning, and directional growth responses. Among these, halotropism—the bending of roots away from high‐salinity zones—enables roots to avoid toxic salt conditions (Galvan‐Ampudia et al. 2013). Understanding the mechanisms underlying halotropism is therefore important for improving crop resilience under saline environments.

Halotropism integrates auxin‐dependent and auxin‐independent pathways. Asymmetric auxin distribution across the root tip, mediated primarily by the efflux carrier PIN2 and influx carrier AUX1, establishes directional growth away from saline patches (Galvan‐Ampudia et al. 2013; van den Berg et al. 2016; Zheng et al. 2024). Abscisic acid triggers phosphorylation of microtubule‐associated proteins, promoting microtubule reorientation and anisotropic cell expansion, facilitating rapid curvature in response to salt gradients (Yu et al. 2022). These findings highlight the multilayered regulatory architecture of halotropism, where hormonal, cytoskeletal and membrane‐trafficking components converge to fine‐tune directional growth.

Hydrotropism, the directional growth of roots toward regions of higher water availability, often counteracting gravitropism (Wexler et al. 2024), provides a framework for understanding potential mechanisms underlying halotropism. Two genes have been shown to be indispensable for hydrotropism: *MIZ1*, which is not required for halotropism (Yu et al. 2022), and *MIZ2/GNOM* (hereafter referred to as *MIZ2*), a guanine nucleotide exchange factor for ARF small GTPases (ADP‐ribosylation factor‐type G proteins) (Miyazawa et al. 2009), whose potential involvement in halotropic responses has not yet been examined; here, we assess the halotropic response of the *miz2* mutant.

Reactive oxygen species (ROS) function as secondary messengers in roots, linking environmental cues to cellular responses during gravitropism (Joo et al. 2001) and hydrotropism (Krieger et al. 2016). NADPH oxidases, particularly RBOHC (also known as RHD2), produce spatially localised ROS in root epidermal cells, supporting root‐hair elongation (Foreman et al. 2003). RBOHC‐generated ROS form a symmetric domain in the elongation zone, acting as a modulatory signal that restrains excessive root curvature during the hydrotropic response. Loss‐of‐function *rbohC* mutants display exaggerated bending, indicating that RBOHC‐derived ROS serve as a regulatory brake on tropic responses (Krieger et al. 2016).

To investigate halotropism independently of gravitropic input, 7‐day‐old *Arabidopsis* seedlings were exposed to a lateral NaCl gradient in a split‐agar system under static conditions or continuous clinorotation (Figure 1a, Supporting Information S1: Figure S1a,b). Static roots exhibited moderate halotropic bending away from salt (20.6° ± 5.4°), whereas clinorotation enhanced curvature (35.7° ± 4.4°), with minimal basal curvature under control conditions (Figure 1b). While not statistically significant, the modest increase in root‐elongation rate under clinorotation (Supporting Information S1: Figure S1c) may slightly accelerate the halotropic response; however, this effect is minor and cannot account for the magnitude of the enhanced bending.

**Figure 1:**
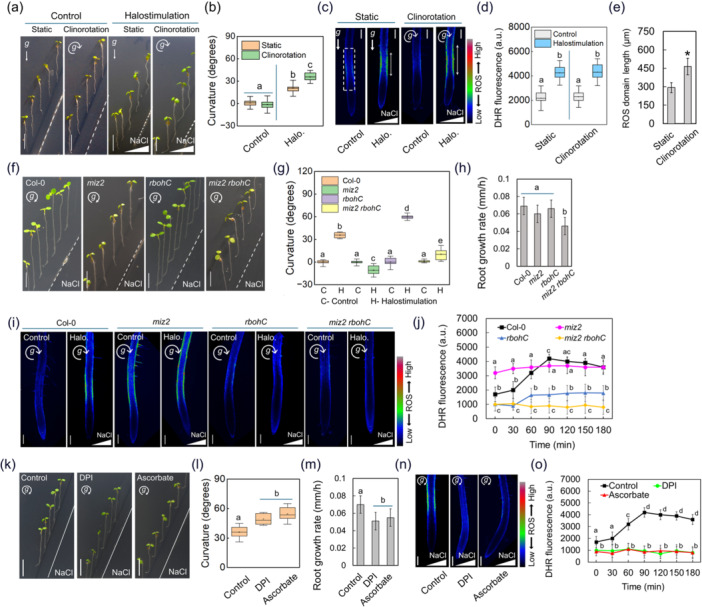
Phenotypic and ROS dynamics during halostimulation under clinorotation. Representative images and quantification of root‐tip curvature and elongation rates (a, b, f–h, k–m) or DHR‐stained ROS levels and domain length (c–e, i, j, n, o) in 7‐day‐old *Arabidopsis* seedlings (indicated genotypes) after 12 h or 90 min of halostimulation, respectively. Assays were performed under static or clinorotation conditions, with or without ascorbate or DPI. *p < 0.01. Scale bars = 0.5 cm (phenotype) and 100 µm (ROS). Quantitative plots show means, interquartile ranges, and SD (see Supporting Information S1).

ROS dynamics during halotropism were examined using the H_2_O_2_‐sensitive probe DHR (see Supporting Information S1: Materials and Methods). Confocal imaging revealed a symmetric ROS domain in the elongation zone, peaking 400–700 µm from the root tip (Figure 1c–e). Static lateral salt exposure increased ROS from 2170 ± 505 a.u. to 4250 ± 443 a.u., whereas clinorotation slightly increased it further to 4310 ± 550 a.u. The longitudinal length of the domain expanded from 293 ± 40 µm under static conditions to 565 ± 67 µm under clinorotation, showing that reduced gravitropic input broadens the ROS field. Exposure to vertical or uniform NaCl did not induce similar ROS patterns (Supporting Information S1: Figures S2, S3), demonstrating that formation of the domain requires directional lateral stimulation.

The role of RBOHC in halotropism was assessed using loss‐of‐function mutant. *rbohC* roots exhibited exaggerated bending under lateral salt exposure (59.7° ± 6.3°) compared to the wild‐type (34.9° ± 8.1°), while elongation rates were comparable to those of the wild‐type (Figure 1f–h). DHR imaging confirmed the absence of ROS accumulation in *rbohC* (Figure 1i,j), demonstrating that RBOHC is required to generate the elongation‐zone ROS domain that restrains excessive curvature. In contrast, *rbohD* mutants displayed wild‐type bending responses (Supporting Information S1: Figure S4), consistent with a specific role for RBOHC in localised ROS production during halotropism and its function in hydrotropism (Krieger et al. 2016).

Chemical modulation of ROS further supported this regulatory model. Treatment with 0.5 mM ascorbate or 1 µM diphenyleneiodonium (DPI), an inhibitor of NADPH oxidase (Foreman et al. 2003), enhanced wild‐type curvature (54.6° ± 8.2° and 51.3° ± 7.2°, respectively) while slightly reducing elongation rates (Figure 1k–m). DHR imaging confirmed near‐complete suppression of the ROS domain (Figure 1n,o), indicating that RBOHC‐derived ROS modulate, rather than drive, bending magnitude.

Proper halotropic orientation depends on the spatial confinement of ROS by MIZ2. In contrast to the positive bending observed in the wild‐type, *miz2* roots exhibited negative halotropism (−10.8° ± 6.4°) under clinorotation, with similar elongation rates (Figure 1f–h). DHR imaging revealed an abnormally broad ROS domain in *miz2* (Figure 1i,j), suggesting that MIZ2 restricts the spatial pattern of RBOHC‐mediated ROS production. Chemical reduction of ROS partially restored positive halotropism, with DPI being more effective than ascorbate (Supporting Information S1: Figure S5), indicating that mislocalized ROS contribute to the inverted bending phenotype. Together, these results imply that ROS acts not as the primary halotropic signal but as a fine‐tuning mechanism, potentially modulating the root's resistance to gravitropic input.

A *miz2 rbohC* double mutant clarified the regulatory hierarchy. Under halostimulation, *miz2 rbohC* roots displayed attenuated but consistently positive curvature (10.4° ± 7.1°), contrasting with the negative bending of *miz2* and exaggerated bending of *rbohC* roots. Root‐elongation rates were only slightly reduced (Figure 1f–h). DHR imaging confirmed the absence of ROS accumulation (Figure 1i,j), showing that RBOHC‐derived ROS causes the mislocalized ROS field in *miz2*. This establishes MIZ2 as upstream of RBOHC in controlling ROS distribution and the halotropic response.

Collectively, halotropism involves a symmetric, spatially restricted ROS domain generated by RBOHC and confined by MIZ2. This domain tempers gravitropic input, allowing lateral salt cues to dominate root directional growth. Clinorotation minimises gravitropic opposition and reveals the full amplitude of halotropic bending, while also inducing an expansion of the ROS domain relative to normal gravity. This expansion is sufficient to destabilise the ROS‐based constraint that normally limits curvature, resulting in exaggerated bending. Loss of RBOHC or chemical suppression of ROS similarly removes this restraining mechanism, whereas mislocalized ROS in *miz2* reverses the direction of growth. The *miz2 rbohC* phenotype further confirms that RBOHC‐derived ROS are the principal contributors to the aberrant response.

Together, these findings demonstrate that ROS act as dynamic regulators of root bending, integrating multiple environmental and cellular signals to fine‐tune halotropic responses. We propose that MIZ2 functions to spatially restrict the abundance and activity of RBOHC; in *miz2* roots, the broad ROS signal present even under control conditions (Figure 1i,j) suggests that the loss of MIZ2‐dependent spatial control results in a constitutive, expanded ROS field. Mechanistic parallels with hydrotropism (Krieger et al. 2016) suggest that while these responses are genetically distinct in their requirement for MIZ1, they converge on this shared MIZ2/RBOHC regulatory module. In this framework, the spatial integrity of the symmetric ROS domain is essential to fine‐tune directional growth; its mislocalization in *miz2* destabilises this regulatory ‘brake’, leading to the observed misdirected curvature. MIZ2‐dependent processes, potentially involving vesicle trafficking, could restrict RBOHC activity to discrete plasma membrane domains, ensuring spatial confinement of ROS. This spatial control is essential for precise modulation of elongation‐zone growth and prevents excessive or misdirected curvature.

Understanding halotropic signalling has broad biological and agricultural implications. As soil salinization increasingly threatens crop productivity, identifying mechanisms controlling directional root growth provides targets for enhancing root plasticity and stress resilience. Future studies combining high‐resolution imaging with targeted perturbation of vesicle trafficking will refine the mechanistic model and directly assess localisation dynamics.

## Conflicts of Interest

The authors declare no conflicts of interest.

## Supporting information

Supporting File

## Data Availability

The data that support the findings of this study are available from the corresponding author upon reasonable request.
