# Supplementary material for: RBOHC‐Generated ROS Tune MIZ2/GNOM‐Dependent Root Halotropism in Arabidopsis
Source: Plant Cell Environ. 2026 Jun 19;49(9):7151–4. doi: 10.1111/pce.70681 (PMC13436457; doi:10.1111/pce.70681)
Supplement: Supplementary file 1 [file PCE-49-7151-s001.docx]

**Supplemental material for:**

**RBOHC-Generated ROS Tune MIZ2/GNOM-Dependent Root Halotropism in *Arabidopsis***

Amir Cohen, Matan Franko, Yvonne Kiere, Yonatan Wexler and Doron Shkolnik*

Robert H. Smith Institute of Plant Sciences and Genetics in Agriculture, Faculty of Agriculture, Food and Environment, Hebrew University of Jerusalem, Rehovot 7610001, Israel

* Author for correspondence: [doron.shkolnik@mail.huji.ac.il](mailto:doron.shkolnik@mail.huji.ac.il)

Supporting information –

Statistical and Quantification Parameters for Figure 1

Figs. S1–S5

Materials and Methods

**Statistical and Quantification Parameters for Figure 1**

For root-curvature and DHR fluorescence box plots (Figure 1b, d, g, l), center lines represent the means, bounds indicate the interquartile range (25th and 75th percentiles), and whiskers represent the standard deviation (SD). For line graphs showing time-course fluorescence (Figure 1j, o) and bar plots showing domain length or growth rates (Figure 1e, h, m), data are presented as mean ± SD. Regarding sample sizes and biological replicates for each panel, root curvature and elongation measurements included n = 90 from four independent experiments for (b, l), n = 80 from four independent experiments for (g), and n = 45 from three independent experiments for (h, m). For ROS fluorescence and domain length quantification, sample sizes were n = 60 from three independent experiments for (d, j, o) and n = 30 from three independent experiments for (e). Statistical significance was evaluated using Tukey’s honestly significant difference (HSD) post-hoc test (*P* < 0.001 for b, d, g, h; *P* < 0.01 for j, l, m, o) or a two-tailed Student’s *t*-test (*P* < 0.01 for e). Different letters or asterisks indicate statistically significant differences between treatments. The dashed box in (c) outlines the 300–900 µm root segments from the apex used for DHR fluorescence quantification. The *g* symbol with the arrow indicates the gravity vector under continuous clinorotation, adopting the visual notation introduced by Wexler et al. (2025) and archived as "Standardized Graphical Symbol for Gravity under Continuous Clinorotation" on Zenodo (https://zenodo.org/records/20271491).

**Figure S1. NaCl/split-agar system utilized for root halostimulation under control gravity and continuous clinorotation conditions.**

**(a) Schematic of the NaCl/split-agar assay. Seedlings were placed diagonally on agar solidified with 0.25X MS medium occupying half of the plate area, followed by addition of 200 mM NaCl-containing medium to the other half. Root curvature was measured at specified time points after treatment initiation; g, gravity vector (direction indicated by arrow). (b) Illustration of the rotation setup for clinorotation experiments. Assay plates were mounted on a rotator to minimize gravitropic influence during halostimulation. (c) Root-elongation rate during treatment as described in Fig. 1a. Values represent mean ± SD (n = 30 from three independent biological replicates); ns, not significant relative to control (Student’s *t*-test).**

**Figure S2. ROS signals are not generated in roots in response to vertical halostimulation. (a) Schematic representation of the vertical NaCl/split-agar system. Control plates were prepared by attaching half-plate medium without NaCl. (b) Quantification of root-tip curvature in five-day-old wild-type (Col-0) *Arabidopsis* seedlings halostimulated (Halo.) for 12 h, either in a static vertical position (as shown in panel a) or under continuous clinorotation. Values represent mean ± SD (n = 45 from three independent experiments). (c) Representative DHR-staining images of roots from control and halostimulated seedlings after 90 min of treatment. Images were pseudo-colored to indicate relative ROS levels. Scale bar = 100 µm; g, gravity vector (direction indicated by arrow). (d) Quantification of DHR fluorescence intensity in 300–900 µm segments from the root apex (dashed box in panel c; a.u., arbitrary units). Values represent mean ± SD (n = 45 from three independent experiments). No significant differences were detected among treatments (Tukey’s HSD, *P* > 0.05).**

### ****Figure S3. Uniform salt exposure does not induce ROS signal in the root-elongation zone.** (a) Schematic representation of the experimental setup used to apply uniform salt stress to entire roots. (b) Representative DHR-staining of roots from five-day-old wild-type (Col-0) *Arabidopsis* seedlings exposed to 0, 25, or 50 mM NaCl for 30 min. Scale bar = 100 µm. (c) Quantification of DHR fluorescence intensity in the elongation zone (as indicated in panel b; a.u., arbitrary units). Bars represent mean ± SD (n = 30 from three independent experiments). No significant differences were detected among treatments (Tukey’s HSD, *P* > 0.05). Uniform salt exposure did not induce ROS accumulation in this region, in contrast to the localized signal observed under halostimulation.**

**Figure S4. RBOHD is not required for the halotropically induced bending response.**

(a) Seven-day-old wild-type (Col-0) and *rbohD* mutant *Arabidopsis* seedlings exposed to halostimulation (Halo.) in a NaCl/split-agar system for 12 h under continuous clinorotation. Representative images shown *g*, gravity vector (under clinorotation**),** scale bar = 0.5 cm. (b) Quantification of root-tip curvature following treatment as in (a). Box plots show interquartile range; lines, means; whiskers, SD (n = 60 from three independent experiments). Different letters indicate significant differences (Tukey’s HSD, *P* < 0.01). (c) Root-elongation rate calculated from primary root length at the start and end of halostimulation. Values represent mean ± SD (n = 30 from three independent experiments); ns, no significant difference relative to Col-0 (Student’s *t*-test).

**Figure S5. DPI and ascorbate affect *miz2* root halotropism**

### (a) Five-day-old wild-type (Col-0) and *miz2* mutant *Arabidopsis* seedlings exposed to halostimulation in a NaCl/split-agar system for 12 h under continuous clinorotation, with control, 0.5 mM ascorbate, or 1 µM DPI treatments. Representative images shown; *g*, gravity vector (under clinorotation**),** scale bar = 0.5 cm. (b) Quantification of root-tip curvature following the treatments described in (a). Box plots show interquartile range; lines, means; whiskers, SD (n = 60 from three independent experiments). (c) Root-elongation rate calculated from primary root length before and after 12 h of treatment. Values represent mean ± SD (n = 45 from three independent experiments); ns, no significant difference relative to Col-0 (Student’s *t*-test).

**Materials and Methods**

**Plant Material and Growth Conditions**

*Arabidopsis thaliana* (ecotype Col-0) and its mutant lines *miz2* (*gnom*^G2852A^) (Miyazawa et al., 2009), *rbohC (rhd2)* and *rbohD* (Miller et al., 2009) were used in this study. Seeds were surface-sterilized and cold-stratified as described previously (Shkolnik & Bar-Zvi, 2008). Germination and seedling growth were carried out on 0.25× Murashige and Skoog (MS) medium (Murashige & Skoog, 1962) solidified with 0.7% (w/v) agar. Square Petri dishes were positioned vertically to allow roots to grow along the medium surface. To obtain seedlings with uniformly straight primary roots and ensure reproducible halotropic responses, seedlings were grown partially embedded in the solidified medium, as previously described (Wexler et al., 2025). Growth conditions were 16-h light/8-h dark cycles at 22°C. *miz2 rbohC* double mutants were generated by crossing the single mutant lines and selfing the F1 progeny; segregating F2 plants were screened to identify individuals carrying both mutant alleles, and homozygous double mutants were confirmed in the F3 generation. Genotyping of *miz2* and *rbohC* alleles was performed by polymerase chain reaction (PCR) using the following primer pairs (5′→3′): MIZ2-Fwd, CTGTTGATGAACCCGTTCTTGC; MIZ2-Rev, GCAAGCTGCAACAAAGATTCAGC; RBOHC-Fwd, CAACTTTGGTGTTAGGATGATCCATAGCTATACG; RBOHC-Rev, CTTCCCGAAAGTCTTGATTGATGGTCC. PCRs were carried out using Q5 High-Fidelity DNA Polymerase (Takara, Japan) according to the manufacturer’s instructions, with an annealing temperature of 60^o^C. For *miz2*, the PCR product was subjected to Sanger sequencing to identify the G2852A point mutation. For *rbohC*, presence of the insertion was inferred from the absence of a PCR product, whereas clear amplification was observed in the wild-type.

**Halostimulation System under Continuous Clinorotation**

To expose roots to a lateral NaCl gradient (halostimulation) while minimizing gravitropic influence, we adapted the split-agar method originally developed for hydrostimulation by replacing sorbitol with NaCl (Takahashi et al., 2002; Krieger et al., 2016; Yu et al., 2022). Five- or seven-day-old seedlings were positioned on control agar-solidified medium occupying half of a 90 mm Petri dish, with root tips placed 5 mm from the medium edge. The other half of the plate was filled with medium supplemented with NaCl (200 mM), allowing gradual diffusion toward the roots (Fig. **S1**). Plates were sealed with parafilm and mounted on a benchtop rotator (ELMI Intelli-Mixer™ RM-2L) rotating at 9 RPM to achieve continuous clinorotation during the assay. For chemical treatments, seedlings were placed on medium containing ascorbic acid (0.5 mM in DDW) or diphenyleneiodonium (DPI; 1 μM in DMSO) 1 h prior to halostimulation. Halostimulation was then initiated by adding the NaCl-containing half of the split-agar system to the medium. Seedlings were imaged using a Nikon D7100 camera with an AF-S DX Micro NIKKOR 85 mm f/3.5G ED VR lens (Nikon, Tokyo, Japan), and root-tip curvature was quantified using ImageJ software (<https://imagej.nih.gov/ij/>).

**Confocal Microscopy and ROS Detection**

ROS accumulation in roots was visualized using the H₂O₂-sensitive probe dihydrorhodamine 123 (DHR), as previously described (Gomes et al., 2005; Krieger et al., 2016). Briefly, immediately following halotropic stimulation, seedlings were immersed in 86.5 μM DHR (0.003% w/v; Sigma-Aldrich) prepared in phosphate-buffered saline (PBS, pH 7.4) for 2 or 5 min. After staining, seedlings were briefly rinsed in PBS to remove excess dye and mounted in PBS on glass slides. Confocal imaging was performed using a Leica SP8 laser-scanning confocal microscope (<https://www.leica-microsystems.com/products/confocal-microscopes/p/leica-tcs-sp8>) equipped with a 10× air objective. Imaging settings were kept constant across all samples to allow quantitative comparison. Excitation was at 488 nm (2% laser power), and emission was collected between 519 and 560 nm. Detector master gain was maintained between 670 and 720 (instrument-specific arbitrary units), with digital gain set to 1. Images were acquired using identical pinhole size, scan speed, and resolution parameters for all treatments. Fluorescence intensity was quantified as mean gray value using ImageJ/Fiji. For length measurements, ROS domains were defined manually using segmented region-of-interest tools, applying the same thresholding criteria across samples. Confocal images were pseudo-colored (RGB LUT) in ImageJ for visualization purposes only, without altering raw pixel values.

**Statistical Analysis and Data Presentation**

Data were analyzed using OriginPro 2024 (OriginLab Corporation) and Microsoft Excel 2021 with the Analysis ToolPak add-in. Graphs and summary statistics were generated using these software packages, and all statistical tests are indicated in the figure legends or main text.

**Accession numbers**

Accession numbers of the major genes investigated in this research are: *RBOHC,* AT5G51060; MIZ2/*GNOM*, AT1G13980.

**References**

Gomes A., Fernandes E., Lima J.L.F.C. 2005 Fluorescence probes used for detection of reactive oxygen species. *J Biochem Biophys Methods* 65: 45–80

Krieger G, Shkolnik D, Miller G, Fromm H 2016 Reactive oxygen species tune root tropic responses. *Plant Physiol* 172:1209-1220

Miller G., Schlauch K., Tam R., Cortes D., Torres M.A., Shulaev V., Dangl J.L., Mittler R. 2009 The plant NADPH oxidase RBOHD mediates rapid systemic signaling in response to diverse stimuli. *Sci Signal* 2: ra45

Miyazawa Y., Takahashi A., Kobayashi A., Kaneyasu T., Fujii N., Takahashi H. 2009 GNOM-mediated vesicular trafficking plays an essential role in hydrotropism of *Arabidopsis* roots. *Plant Physiol* 149: 835–840

Murashige T., Skoog F. 1962 A revised medium for rapid growth and bio assays with tobacco tissue cultures. *Physiol Plant* 15: 473–497

Shkolnik D., Bar-Zvi D. 2008 Tomato ASR1 abrogates the response to abscisic acid and glucose in Arabidopsis by competing with ABI4 for DNA binding. *Plant Biotechnol J* 6: 368–378

Takahashi N., Goto N., Okada K., Takahashi H. 2002 Hydrotropism in abscisic acid, wavy, and gravitropic mutants of *Arabidopsis thaliana*. *Planta* 216: 203–211

Wexler Y., Kiere Y., Sobol G., Nuriel R., Azoulay-Portal S., Cohen A., Toporik H., Pasmanik-Chor M., Finkler A.., Shkolnik D. 2025 Modulation of Root Hydrotropism and Recovery From Drought by *MIZ1*-like Genes in Tomato. *Plant Cell Environ* 48: 2739–2754

Yu B., Zheng W., Xing L., Zhu J.K., Persson S., Zhao Y. 2022 Root twisting drives halotropism via stress-induced microtubule reorientation. *Dev Cell* 57: 2412-2425.e6
